# Association of HLA-DPA1, HLA-DPB1, and HLA-DQB1 Alleles With the Long-Term and Booster Immune Responses of Young Adults Vaccinated Against the Hepatitis B Virus as Neonates

**DOI:** 10.3389/fimmu.2021.710414

**Published:** 2021-08-18

**Authors:** Wen-Chang Wang, Yu-Shiang Lin, Yin-Fan Chang, Chih-Ching Yeh, Chien-Tien Su, Jin-Shang Wu, Fu-Hsiung Su

**Affiliations:** ^1^The Ph.D. Program for Translational Medicine, College of Medical Science and Technology, Taipei Medical University, Taipei, Taiwan; ^2^Cancer Center, Wan Fang Hospital, Taipei Medical University, Taipei, Taiwan; ^3^School of Public Health, College of Public Health, Taipei Medical University, Taipei, Taiwan; ^4^Department of Clinical Laboratory, the First Affiliated Hospital, School of Medicine, Xi’an Jiaotong University, Xi’an, China; ^5^Department of Family Medicine, National Cheng Kung University Hospital, College of Medicine, National Cheng Kung University, Tainan, Taiwan; ^6^Department of Public Health, College of Public Health, China Medical University, Taichung, Taiwan; ^7^Master Program in Applied Epidemiology, College of Public Health, Taipei Medical University, Taipei, Taiwan; ^8^Department of Family Medicine, Taipei Medical University Hospital, Taipei, Taiwan; ^9^Department of Family Medicine, National Cheng Kung University Hospital, Douliou Branch, College of Medicine, National Cheng Kung University, Yunlin, Taiwan; ^10^Department of Family Medicine, College of Medicine, National Cheng Kung University, Tainan, Taiwan; ^11^Department of Family Medicine, Cardinal Tien Hospital, Fu Jen Catholic University, New Taipei City, Taiwan; ^12^School of Medicine, College of Medicine, Fu Jen Catholic University, New Taipei City, Taiwan

**Keywords:** hepatitis B vaccine, human leukocyte antigen, booster, HBV serology, immunogenic response

## Abstract

The neonatal hepatitis B vaccination (HBVac) was implemented 35 years ago in Taiwan, but many vaccinees exhibit inadequate long-term vaccine-induced seroprotective hepatitis B surface antibody (anti-HBs) levels. We investigated the association of the human leukocyte antigen (HLA) alleles (DPA1, DPB1, DQA1, and DQB1) with the long-term immunological response to the neonatal HBVac and adolescent booster HBVac in a Taiwanese cohort. We divided 281 Han students (median age 22, age range 17–29 years) into the following groups: (1) Group A (*n* = 61): anti-HBs titer ≥ 10 mIU/mL at the beginning of the study; (2) Group B (*n* = 75): anti-HBs level > 1000 mIU/mL after the first booster; (3) Group C (*n* = 37): anti-HBs level < 10 mIU/mL after the first booster; and (4) Group D (*n* = 5): anti-HBs level < 10 mIU/mL after three boosters. DQA1, DQB1, DPA1, and DPB1 typing of the participants was performed using sequence-specific oligonucleotides. Associations of HLA alleles and haplotypes with effects on neonatal HBVac and booster HBVac were examined through logistic regression analysis and Fisher’s exact test. A false discovery rate-based measure of significance, the q-value, was used for multiple comparisons, and an association was considered significant if the corresponding q-value was < 0.1. DPA1 alleles were associated with the long-term immunological response to the neonatal HBVac. The estimated odds ratio (OR) of the lack of HBV protective immunity when carrying an additional DPA1*01 and DPA1*02 was 0.36 [95% confidence interval (CI) = 0.17–0.76, p = 0.0076] and 2.39 (95% CI = 1.17–4.87, p = 0.016), respectively. DPB1 and DQB1 alleles were associated with a response to the adolescent booster vaccination. The estimated ORs of being nonresponsive to the first booster when carrying an additional DPB1*05 and DQB1*02 were 2.11 (95% CI = 1.13–3.93, p = 0.019) and 3.73 (95% CI = 1.43–9.71, p = 0.0070), respectively. All DPB1*03 carriers responded to the first booster (p of Fisher’s exact test = 0.0045). In our study, we discovered that HLA-DPA1 was primarily associated with the long-term response of primary infantile HBVac, and HLA-DPB1 and HLA-DQB1 exhibited associations with the HBV booster vaccination.

## Introduction

Hepatitis B virus (HBV) infection is a major cause of chronic hepatitis, liver cirrhosis, and hepatocellular carcinoma globally (1) and continues to be a serious public health threat worldwide. Globally, More than 250 million people have had chronic HBV infection, with the majority living in Asia or Africa (2). Maternal transmission during the perinatal period and horizontal transmission in early life constitute the main transmission routes of HBV infection (3). To combat global HBV infection, the World Health Organization (WHO) recommended in 1997 that the HBV vaccination (HBVac) be incorporated into routine infant and childhood immunization programs (4). By the end of 2019, 189 member states of the WHO have implemented these infant HBVac programs. The global coverage rate of the three doses of HBV vaccine among infants has reached 85% worldwide (5). The dramatic effect of neonatal HBV immunization on the decrease in the prevalence of the HBV surface antigen (HBsAg) among children has been reported by many countries including China (1%), South Korea (0.12%), Iran (0.6%), Colombia (0.5%), Italy (0.6%), Saudi Arabia (0.3%), and Canada (0.3%) (3). Taiwan was one of the first countries to implement national neonatal HBVac in 1984. Prior to this, approximately 15% to 20% of adults tested positive for the HBsAg (6, 7). Since the introduction of universal neonatal HBVac, the number of chronic HBV infections in Taiwanese adolescents has decreased substantially (8).

Despite the proven immunogenicity of the HBVac, adequate long-term vaccine-induced seroprotective hepatitis B surface antibody (anti-HBs) level has not been observed in a significant proportion of HBV vaccinees, which may indicate a loss of immunological memory against HBsAg (9, 10). The WHO states that no evidence has been provided to support the requirement of booster dose of the HBVac in a person who received the complete series of HBVacs in the past (such as in infancy or adolescence) (11). However, the Advisory Committee on Immunization Practices (ACIP) within the US Centers for Disease Control and Prevention recommends that healthcare personnel (HCP) with evidence of having previously been administered a proper series of HBVacs but who test negative for anti-HBs must receive one “booster” dose of the HBVac and be retested 1 to 2 months later. If the HCP retest is negative after one booster dose, they must complete a second series of HBVacs and be tested for the anti-HBs again 1 to 2 months after the last dose (12).

Despite the recommendations of the ACIP and WHO, some evidence has reported that breakthrough and chronic HBV infections have been observed in adolescent vaccinees who had received a complete cycle of neonatal HBV vaccines (10, 13–16). Numerous factors including age, sex, smoking, extreme obesity, ethnicity, and immunological tolerance can influence the immune response to the HBV vaccine (17). Newport et al. reported that approximately 77% of the variation in anti-HBs response to the HBVac was attributable to genetic factors (18). Other studies conducted on mono- and dizygotic twins have indicated that genetic factors may account for nearly 60% of immune responsiveness to the HBVac, with human leukocyte antigen (HLA) genes being the major contributors (19).

A major role of HLAs is to regulate immune responses by processing and presenting protein antigens to T cells. HLA genes are the most polymorphic in the human genome. The varying polymorphism can generate disparities in HLA molecule processing and antigen–peptide presentation ability, consequently influencing the humoral immune response to HBV antigens (20). Many studies have also indicated that several HLA alleles are closely linked to the immunological response to the HBVac (21–23). Hence, HLA is also hypothesized to play a key role in responsiveness to the HBVac (24).

Research data has identified considerable ethnic differences in HLA. A variety of HLA class I and class II alleles and HLA extended haplotypes increased or decreased in the nonresponse of individuals from different ethnic backgrounds (25). The possible associations of human leukocyte antigen class I (HLA-A, B, and C) and human leukocyte antigen class II (HLA-DR) loci with the antibody response to the HBVac have been extensively investigated internationally (20, 26–28). Taiwanese studies have also identified variants in HLA-A, -B, and -DR that correlated with the response to the booster HBVac (23, 29).

In this study, we investigated the association of HLA-DPA1, HLA-DPB1, HLA-DQA1, and HLA-DQB1 alleles among a young Taiwanese population with anti-HBs persistence and who have observably lost immune memory to the neonatal HBV vaccine. We also compared the HLA-DPA1, HLA-DPB1, HLA-DQA1, and HLA-DQB1 alleles in the high HBVac booster to a single-booster dose with (1) nonresponders to the single-booster dose and (2) nonresponders to the three-booster dose of the same HBV vaccine.

## Materials and Methods

### Study Population

From November 2015 to November 2016, 281 university health science student volunteers of Han ethnicity who had received a complete cycle of HBV vaccines during infancy (30) were recruited from two universities (in the north and south of the island). To avoid the small portion of nonresponders to the HBV booster who may have occult HBV infection and therefore do not respond to HBVacs, students with positive hepatitis B core antibody (anti-HBc) were excluded from the study. All students were recruited during health check-ups that are part of their preclinical rotation and who had a negative HBsAg and anti-HBc serum status. Students who were unable to provide HBVac records (either a vaccination document or verbal confirmation from their parents), were cigarette smokers or betel nut chewers, were pregnant, had a medical history of chronic illness, or had an allergy to yeast or the HBV vaccine were excluded from the study. All participants provided written informed consent, and this study was approved by the Joint Institutional Review Board of Taipei Medical University (N201511029) and Institutional Review Board of National Cheng Kung University Hospital (B-ER-106-006).

### Serum HBV Markers

During the initial health examination, serum levels of the HBsAg, anti-HBs, and anti-HBc were determined using a commercially available enzyme immunoassay kit (Elecsys 2010 System; Roche Diagnostics, Mannheim, Germany) in the northern university. Samples with an anti-HBs titer of ≥10 and <10 mIU/mL were interpreted as reactive and nonreactive, respectively. The measurement range was defined as 2 to 1000 mIU/mL, and values below the limit of detection were reported as <2 mIU/mL. Values above the measurement range were reported as >1000 mIU/mL. For the interpretation of the serum anti-HBc level, samples with a cutoff index > 1 were designated nonreactive and do not require further testing. Samples with a cutoff index ≤ 1 were reactive and were subsequently retested in duplicate. If the results of the follow-up test were nonreactive in both cases, the sample was judged negative for the anti-HBc. The detection limit of the anti-HBc was ≤ 0.8 WHO IU/mL. HBsAg samples with a cutoff index < 0.90 were nonreactive and judged negative for the HBsAg; no further testing was required. HBsAg samples with a cutoff index ≥ 1 were considered reactive, and those with a cutoff index in the range of ≥0.90 to <1 were considered borderline in the Elecsys HBsAg II assay. All initially reactive or borderline samples were redetermined using the HBsAg II assay. If cutoff index values <0.90 were recorded in both cases, the sample was judged negative for the HBsAg. Initially reactive or borderline samples with cutoff index values of ≥0.90 in either of the redeterminations were considered repeatedly reactive. Repeatedly reactive samples were investigated using an independent neutralization test (Elecsys HBsAg Confirmatory Test).

For the southern university, serum levels of the HBsAg, anti-HBs, and anti-HBc were determined using SURASE B-96 (tetramethylbenzidine solution [TMB]), ANTISURASE B-96 (TMB), and ANTICORASE B-96 (TMB) enzyme immunoassay diagnostic kits (General Biological Corporation, Hsinchu, Taiwan). Specimens with an absorbance value less than the cutoff value in the SURASE B-96 assay were nonreactive and considered negative for the HBsAg. Specimens with an absorbance value greater than or equal to the cutoff value were retested in duplicate. For a reactive repeat reaction, the sample determination was validated using confirmatory reagents for the HBsAg. Only confirmed positive specimens were judged to contain the HBsAg. A specimen with an absorbance value less than the 0.9 X cutoff value was considered nonreactive for anti-HBs by using the ANTISURASE B-96 manufacturer criteria. A specimen with an absorbance value greater than the 1.1 X cutoff value was considered reactive for the anti-HBs. Specimens with an absorbance value within the retest range (cutoff value ± 10%) were tested in duplicate. The analytical sensitivity was determined to be 3.6 mIU/mL of the anti-HBs for the ANTISURASE B-96 assay. The standard cutoff for 10 mIU/mL of the anti-HBs was approximately 1.60. A specimen with an absorbance value greater than the 1.1 X cutoff value of the ANTICORASE B-96 assay was considered negative for the anti-HBc. A specimen with an absorbance value less than the 0.9 X cutoff value was considered positive for the anti-HBc. Specimens with an absorbance value within the retest range (cutoff value ± 10%) were tested in duplicate.

The detection limit of the anti-HBs in the southern university (3.6 mIU/mL) was slightly lower than that of (2 mIU/mL) the northern university during the initial health examination. However, our experiment design followed the current WHO standards, in which the value of <10 mIU/mL is classified as nonreactive and requiring a HBV booster dose. Thus, such limitations are unlikely to affect the design and analysis of this study.

For the subsequent HBV-vaccine booster dose, a 20 µg/mL/vial recombinant HBV vaccine (Engerix-B; GlaxoSmithKline Biologicals, Rixensart, Belgium) was administered. Serum samples from both universities were stored in a single shared laboratory. Serum level anti-HBs following the HBV booster dose were analyzed using a commercially available Elecsys anti-HBs II (2010 System; Roche Diagnostics, Mannheim, Germany) assay to detect the HBV booster effect.

### Design of the Study Cohort

**Figure 1:**
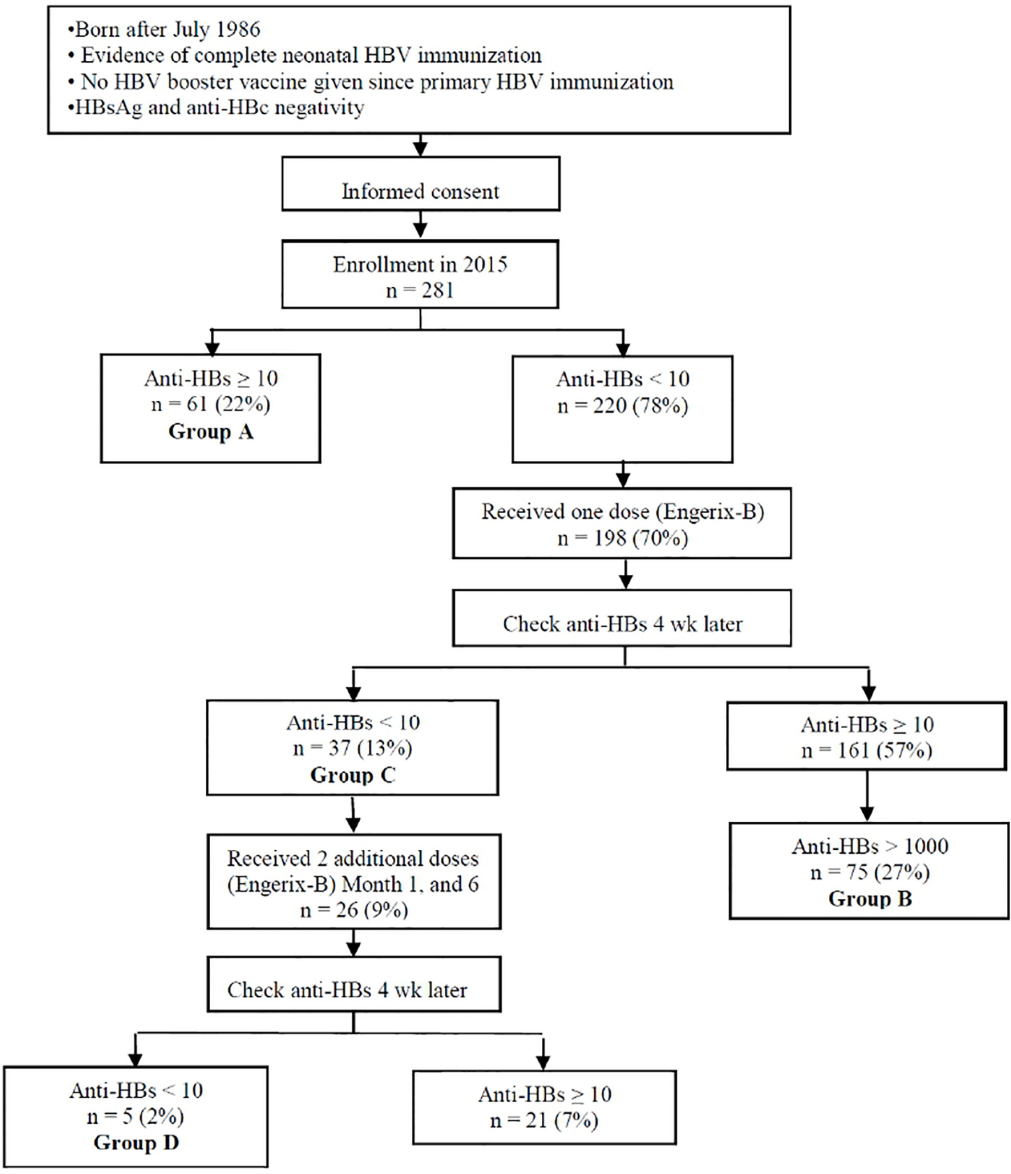
Study flow chart. Group A: anti-HBs titer ≥ 10 mIU/mL when entering the study; Group B: anti-HBs level > 1000 mIU/mL after the first booster; Group C: anti-HBs level < 10 mIU/mL after the first booster; and Group D: anti-HBs level < 10 mIU/mL after all HBV boosters.

**Figure 1** presents the design of the study. Participants were divided into the following four groups: Group A with an anti-HBs titer of ≥10 mIU/mL at the beginning of the study; Group B with an anti-HBs level of >1000 mIU/mL following the first booster; Group C with an anti-HBs level of <10 mIU/mL following the first booster; and Group D with an anti-HBs level of <10 mIU/mL following three HBV booster doses. The main association analyses were based on two group to group samples collected from the study population. (1) In Set A, we compared probands with anti-HBs persistence (Group A) to those that had lost immune memory (Group C) to the neonatal HBV vaccine. (2) In Set B, we compared single-booster dose high responders (Group B) to single-booster dose nonresponders (Group C) and three-booster dose nonresponders (Group D) to the same HBV vaccine, respectively.

### Protocol of the HBV-Vaccine Booster Doses

HBV protective immunity in 281 students who had been neonatally vaccinated with a complete cycle of HBV vaccines (33 were four-dose plasma-derived recipients, and 248 were three-dose recombinant HBV vaccine recipients at 0, 1, 2, and 12 months and at 0, 1, and 6 months of age, respectively) was assessed between 2015 and 2016. Those who lacked HBV protective immunity (anti-HBs < 10.0 mIU/mL) received a booster of the 20 µg/mL/vial Engerix-B recombinant HBV vaccine through intramuscular injection into the deltoid muscle. Anti-HBs titers were measured 4 weeks later. For participants whose serum anti-HBs level remained less than 10.0 mIU/mL, two doses of the recombinant HBV vaccine (20 µg/mL/vial each) were administered (in the second and sixth month, respectively). Anti-HBs titers were measured again at the end of the seventh month following the first HBV booster vaccine administration.

### HLA Typing

Genomic DNA was extracted from the ethylene diaminetetraacetic acid (EDTA)-containing peripheral blood samples by using the QIAamp DNA Blood Mini Kit (Qiagen, Germany) and was then preserved at −70°C. The HLA-DQA1, HLA-DQB1, HLA-DPA1, and HLA-DPB1 typings were performed using the LIFECODES HLA-DQA1/B1 SSO Typing Kit and LIFECODES HLA-DPA1/B1 SSO typing kit (Immucor GTI Diagnostics, Inc. WI, USA). Briefly, a polymerase chain reaction (PCR) mixture was prepared using 6 μL of the LIFECODES Master Mix, 80 ng of genomic DNA, and 0.2 μL of Taq polymerase in a final volume of 20 μL and then treated with the following: denaturation at 95°C for 3 min; 40 cycles of amplification (12 cycles: 95°C for 15 seconds (s), 60°C for 30 s, and 72°C for 30 s; and 28 cycles: 95°C for 10 s, 63°C for 30 s, and 72°C for 30 s); 1 cycle extension at 72°C for 2 min. The PCR mixture is remained at 4°C until the next step. Hybridization was performed under the following conditions: 97°C for 2 min, 47°C for 10 min, and 56°C for 8 min with 15 μL of probe mix to 5 μL of locus-specific polymerase chain reaction products. The hybridizing samples were diluted with 170 µL of the 1:200 prepared dilution buffer and analyzed within 30 min using the Luminex 200 system (Luminex Corp., Austin, TX, USA). The probe hit pattern was compared with the common and well-documented HLA alleles by using the MatchIT DNA program (Immucor GTI Diagnostics, Inc. WI, USA).

### Statistical Analysis

This study investigated the associations of HLA alleles and haplotypes with the long-term immunological response to the primary neonatal HBVac and adolescent HBV booster vaccination. For each of the four HLA loci, association tests were conducted only for alleles with frequencies larger than 0.05 among the whole study population. In the Taiwanese population, as reported in Huang et al. (2020), HLA-DQ and HLA-DP loci were located in different haplotype blocks (31); therefore, haplotype association analyses for DQA1-DQB1 and DPA1-DPB1 loci were conducted individually. The phased haplotypes of study participants on DQA1-DQB1 and DPA1-DPB1 were estimated separately using PHASE v2.1 software (32). For both DQA1-DQB1 and DPA1-DPB1 loci, association tests were implemented only for haplotypes with frequencies larger than 0.03 among the whole study population.

Association analyses of the long-term effect of the HBV vaccine were conducted by comparing participants with anti-HBs persistence (Group A in **Figure 1**) with those who have obviously lost immune memory to the neonatal HBV vaccine (Group C in **Figure 1**). For a specific allele or haplotype, the association was examined separately under additive and dominant genetic models through logistic regression analyses. In each analysis, the odds ratio (OR) of the lack of HBV protective immunity for carriers of the examined allele or haplotype under a specific genetic model was calculated alongside the corresponding 95% confidence interval (CI) and p value, which were adjusted for age and sex. Associations were also examined by implementing Fisher’s exact tests; these tests and the logistic regression analyses were implemented using R software.

Analyses of associations with response to the HBV-vaccine booster were conducted through a comparison of single-booster dose high responders (Group B in **Figure 1**) with nonresponders (Group C in **Figure 1**). For a specific allele or haplotype, the association with the risk of being a nonresponder was also examined by implementing logistic regression analyses and Fisher’s exact tests under different genetic models.

To manage the multiple testing problem, QVALUE software was used to calculate a q-value for each test to determine the false discovery rate-based measure of significance (33). In this study, an association was considered significant under multiple comparisons if the corresponding q-value was less than 0.1. Following the application of this q-value threshold, the expected proportion of false positive results among the declared associations was 10%.

Furthermore, when an HLA allele was observed to be associated with effects on the HBV vaccine in the comparison of Group A with Group C or Group B with Group C, further association analyses for this allele were conducted through a comparison of Group B with nonresponders to the three-booster dose of the same HBV vaccine (Group D in **Figure 1**). The association of the examined allele with the risk of being a three-booster dose nonresponder was also examined using logistic regression analyses and Fisher’s exact tests under different genetic models.

## Results

As detailed in **Figure 1** and **Table 1**, the study population consisted of 281 individuals with an age range of 17 to 29 years, among whom 130 (46%) were men. HLA typing was performed for all participants, and the distribution of allele frequencies is summarized in **Supplementary Table S1**. Specifically, among the whole study population, 16 HLA alleles with frequencies larger than 0.05 were observed (01 and 02 in DPA1, 02, 03, 04, 05, and 13 in DPB1, 01, 02, 05, and 06 in DQA1, and 02, 03, 04, 05, and 06 in DQB1), and associations of these alleles with the long-term effect of the HBV vaccine and HBV-vaccine booster response were examined.

**Table 1:** Study population characteristics.

Sample group	Group size	Proportion of male participants	Age
	n	n (%)	Median [range], years
Whole study population	281	130 (46)	22 [17, 29]
Anti-HBs ≥10 mIU/mL before first booster	61	26 (43)	23 [21, 28]
Anti-HBs <10 mIU/mL before first booster	220	104 (47)	22 [17, 29]
Participants receiving first booster	198	100 (51)	22 [17, 29]
Anti-HBs ≥10 mIU/mL after first booster	161	74 (46)	22 [19, 29]
Anti-HBs <10 mIU/mL after first booster	37	26 (70)	23 [17, 29]
Participants receiving all boosters	26	21 (81)	23 [17, 28]
Anti-HBs ≥10 mIU/mL after all boosters	21	17 (81)	23 [17, 28]
Anti-HBs <10 mIU/mL after all boosters	5	4 (80)	23 [21, 27]

As depicted in **Figure 1**, the numbers of participants possessing and lacking HBV protective immunity in the whole study population were 61 and 220, respectively. These 61 with an anti-HBs titer of ≥10 mIU/mL at the beginning of the study were designated Group A. Subsequently, 198 participants lacking HBV protective immunity received a single-dose booster of the HBV vaccine. Among them, 75 of the 161 responders had an anti-HBs level of >1000 mIU/mL and were designated Group B; 37 did not respond to the first booster (i.e., with an anti-HBs level of <10 mIU/mL) and were classified as Group C. For the first booster, the proportion of men among the nonresponders was higher than that among the responders (70% *vs.* 46%, **Table 1**). Finally, 26 received the complete three-dose booster of the HBV vaccine, among whom 5 were nonresponders (with an anti-HBs level of <10 mIU/mL) and were designated Group D.

For the 16 HLA alleles with frequencies larger than 0.05, significant associations with the long-term effect or response to the booster were described according to the locus as follows.

The two DPA1 alleles with frequencies larger than 0.05 were DPA1*01 (29.9%) and DPA1*02 (67.4%). As presented in **Table 2**, a comparison of Group A with Group C revealed that both alleles were significantly associated with the long-term effect of the HBV vaccine, which was apparent under the additive model. Specifically, DPA1*01 was associated with a strong long-term effect; the estimated OR for the lack of HBV protective immunity when carrying an additional DPA1*01 was 0.36 (95% CI = 0.17–0.76, p = 0.0076, q = 0.082). Conversely, DPA1*02 was associated with a weak long-term effect; the estimated OR for the lack of HBV protective immunity when carrying an additional DPA1*02 was 2.39 (95% CI = 1.17–4.87, p = 0.016, q = 0.091). Furthermore, as described in **Table 2**, these two DPA1 alleles were associated with response to the HBV-vaccine booster under the additive model. In the comparison of Group B with Group C, the estimated ORs of being a nonresponder to the first booster when carrying an additional DPA1*01 and DPA1*02 were 0.49 (95% CI = 0.22–1.09, p = 0.080 and Fisher’s exact test p = 0.038) and 1.92 (95% CI = 0.92–3.99, p = 0.080 and Fisher’s exact test p = 0.030), respectively. However, these associations were nonsignificant when analyzing multiple comparisons because the corresponding q-values were all greater than 0.1.

**Table 2:** Significant associations of DPA1 alleles with effects of the HBV vaccine.

Examined allele	Model	Genotype*^a^*	Frequency *n* (%)			Group A *vs*. Group C			Group B *vs*. Group C		
(*A*)			Group A	Group B	Group C	OR (95% CI)^b^	p (q)	Fisher’s p (q)*^c^*	OR (95% CI)*^b^*	p (q)	Fisher’s p (q)*^c^*
DPA1*01	ADD	*XX*	26 (42.6)	36 (48)	24 (65)	0.36 (0.17, 0.76)	**0.0076** (0.082)	**0.0059** (0.082)	0.49 (0.22, 1.09)	0.080	0.038 (0.104)
		*AX*	26 (42.6)	32 (43)	13 (35)						
		*AA*	9 (14.8)	7 (9)	0 (0)						
	DOM	*XX*	26 (42.6)	36 (48)	24 (65)	Reference	—		Reference	—	
		*AX+AA*	35 (57.4)	39 (52)	13 (35)	0.38 (0.16, 0.92)	0.032 (0.104)	0.039 (0.104)	0.55 (0.22, 1.36)	0.20	0.11
DPA1*02	ADD	*XX*	10 (16)	9 (12)	1 (3)	2.39 (1.17, 4.87)	**0.016** (0.091)	**0.011** (0.091)	1.92 (0.92, 3.99)	0.080	0.030 (0.104)
		*AX*	26 (43)	34 (45)	13 (35)						
		*AA*	25 (41)	32 (43)	23 (62)						
	DOM	*XX*	10 (16)	9 (12)	1 (3)	Reference	—		Reference	—	
		*AX+AA*	51 (84)	66 (88)	36 (97)	7.16 (0.87, 59.21)	0.068	0.048 (0.104)	5.06 (0.58, 44.54)	0.14	0.16

a XX, noncarrier of the examined allele; AX, participant carrying exactly one examined allele; AA, homozygous carrier of the examined allele.

b Adjusted for age and gender.

c p value of Fisher’s exact test.

ADD, additive model; DOM, dominant model.

Group A: participants with anti-HBs titer ≥ 10 mIU/mL at the beginning of the study.

Group B: participants with anti-HBs level > 1000 mIU/mL after the first booster.

Group C: participants with anti-HBs level < 10 mIU/mL after the first booster.

Q-value is indicated for a p < 0.05 and p value is indicated in bold when the q-value < 0.1.

Among the five DPB1 alleles with frequencies larger than 0.05, DPB1*03 and DPB1*05 were significantly associated with response to the HBV-vaccine booster (**Supplementary Table S2**). As detailed in **Table 3**, all carriers of DPB1*03 were responders to the first booster; the p value obtained using Fisher’s exact test under the dominant model was 0.0045 (q = 0.082). By contrast, DPB1*05 was associated with a high risk of being a nonresponder to the first booster, which was apparent under the additive model (**Table 3**). The estimated OR of being a nonresponder when carrying an additional DPB1*05 was 2.11 (95% CI = 1.13–3.93, p = 0.019, q = 0.091).

**Table 3:** Significant associations of DPB1 alleles with effects of the HBV vaccine.

Examined allele	Model	Genotype*^a^*	Frequency n (%)			Group A *vs.* Group C			Group B *vs.* Group C		
(*A*)			Group A	Group B	Group C	OR (95% CI)*^b^*	p (q)	Fisher’s p (q)*^c^*	OR (95% CI)*^b^*	p (q)	Fisher’s p (q)*^c^*
DPB1*03	ADD	*XX*	56 (92)	62 (83)	37 (100)	N/A	N/A	0.16	N/A	N/A	**0.0058** (0.082)
		*AX*	5 (8)	12 (16)	0 (0)						
		*AA*	0 (0)	1 (1)	0 (0)						
	DOM	*XX*	56 (92)	62 (83)	37 (100)	Reference	—		Reference	—	
		*AX+AA*	5 (8)	13 (17)	0 (0)	N/A	N/A	0.15	N/A	N/A	**0.0045** (0.082)
DPB1*05	ADD	*XX*	14 (23)	29 (39)	6 (16)	1.47 (0.80, 2.72)	0.22	0.30	2.11 (1.13, 3.93)	**0.019** (0.091)	**0.0066** (0.082)
		*AX*	32 (52)	33 (44)	18 (49)						
		*AA*	15 (25)	13 (17)	13 (35)						
	DOM	*XX*	14 (23)	29 (39)	6 (16)	Reference	—		Reference	—	
		*AX+AA*	47 (77)	46 (61)	31 (84)	1.48 (0.50, 4.34)	0.47	0.61	3.01 (1.03, 8.79)	0.044 (0.104)	**0.018** (0.091)

a XX, noncarrier of the examined allele; AX, participant carrying exactly one examined allele; AA, homozygous carrier of the examined allele.

b Adjusted for age and gender.

c p value of Fisher’s exact test.

ADD, additive model; DOM, dominant model.

Group A: participants with anti-HBs titer ≥ 10 mIU/mL at the beginning of the study.

Group B: participants with anti-HBs level > 1000 mIU/mL after the first booster.

Group C: participants with anti-HBs level < 10 mIU/mL after the first booster.

Q-value is indicated for a p < 0.05 and p value is indicated in bold when the q-value < 0.1.

N/A, not available.

In this study, no DQA1 allele was observed to be associated with the long-term effect of the HBV vaccine or response to the HBV-vaccine booster (**Supplementary Table S3**).

Among the five DQB1 alleles with frequencies larger than 0.05, DQB1*02 was significantly associated with a weaker response to the HBV-vaccine booster, indicated using the additive model (**Supplementary Table S4**). In the comparison of Group B with Group C, as presented in **Table 4**, the estimated OR of being a nonresponder to the first booster when carrying an additional DQB1*02 was 3.73 (95% CI = 1.43–9.71, p = 0.0070, q = 0.082). The significant association of DQB1*02 with a weak response to the HBV-vaccine booster was further examined through a comparison of Group B with Group D. As described in **Supplementary Table S5**, the estimated OR of being a nonresponder to the three-booster doses when carrying an additional DQB1*02 was 9.17 (95% CI = 1.63–51.49, p = 0.012 and Fisher’s exact test p = 0.029).

**Table 4:** Significant associations of DQB1 alleles with effects of the HBV vaccine.

Examined allele	Model	Genotype*^a^*	Frequency n (%)			Group A *vs*. Group C			Group B *vs*. Group C		
(*A*)			Group A	Group B	Group C	OR (95% CI)*^b^*	p (q)	Fisher’s p (q)*^c^*	OR (95% CI)*^b^*	p (q)	Fisher’s p (q)*^c^*
DQB1*02	ADD	*XX*	52 (85)	67 (89)	26 (70)	2.14 (0.91, 5.03)	0.079	0.066	3.73 (1.43, 9.71)	**0.0070** (0.082)	**0.0087** (0.082)
		*AX*	8 (13)	7 (9)	9 (24)						
		*AA*	1 (2)	1 (1)	2 (5)						
	DOM	*XX*	52 (85)	67 (89)	26 (70)	Reference	—		Reference	—	
		*AX+AA*	9 (15)	8 (11)	11 (30)	2.37 (0.86, 6.55)	0.096	0.12	4.92 (1.52, 15.95)	**0.0080** (0.082)	**0.016** (0.091)
DQB1*06	ADD	*XX*	36 (59)	46 (61.3)	30 (81)	0.42 (0.18, 1.02)	0.056	0.054	0.43 (0.18, 1.00)	0.050 (0.104)	0.045 (0.104)
		*AX*	23 (38)	25 (33.3)	6 (16)						
		*AA*	2 (3)	4 (5.3)	1 (3)						
	DOM	*XX*	36 (59)	46 (61.3)	30 (81)	Reference	—		Reference	—	
		*AX+AA*	25 (41)	29 (38.6)	7 (19)	0.34 (0.13, 0.91)	0.032 (0.104)	0.028 (0.104)	0.39 (0.14, 1.09)	0.072	0.052

a XX, noncarrier of the examined allele; AX, participant carrying exactly one examined allele; AA, homozygous carrier of the examined allele.

b Adjusted for age and gender.

c p value of Fisher’s exact test.

ADD, additive model; DOM, dominant model.

Group A: participants with anti-HBs titer ≥ 10 mIU/mL at the beginning of the study.

Group B: participants with anti-HBs level > 1000 mIU/mL after the first booster.

Group C: participants with anti-HBs level < 10 mIU/mL after the first booster.

Q-value is indicated for a p < 0.05 and p value is indicated in bold when the q-value < 0.1.

As presented in **Table 4**, DQB1*06 was associated with more favorable effects on the HBV vaccine. For the long-term effect, the estimated OR of the lack of HBV protective immunity for carriers of DQB1*06 compared with noncarriers was 0.34 (95% CI = 0.13–0.91, p = 0.032, and Fisher’s exact test p = 0.028). For response to the HBV-vaccine booster, the estimated OR of being a nonresponder when carrying an additional DQB1*06 was 0.43 (95% CI = 0.18–1.00, p = 0.0497 and Fisher’s exact test p = 0.045). However, these associations were nonsignificant when analyzing multiple comparisons because the corresponding q-values were all greater than 0.1.

The frequencies of haplotypes on DPA1-DPB1 and DQA1-DQB1 loci in the whole study population are summarized in **Supplementary Table S6**. Associations with effects on the HBV vaccine were examined for haplotypes with frequencies larger than 0.03, of which six DPA1-DPB1 haplotypes and eight DQA1-DQB1 haplotypes were identified.

As detailed in **Supplementary Table S7**, among the six DPA1-DPB1 haplotypes, DPA1*02-DPB1*05 and DPA1*01-DPB1*03 were significantly associated with effects on vaccine booster. Specifically, DPA1*02-DPB1*05 was associated with a weaker effect, and the estimated OR of being a nonresponder to the first booster when carrying an additional DPA1*02-DPB1*05 was 2.11 (95% CI = 1.13–3.93, p = 0.019, q = 0.091). Conversely, all carriers of DPA1*01-DPB1*03 were responders to the first booster; the p value of Fisher’s exact test under the dominant model was 0.015 (q = 0.091).

Among the eight DQA1-DQB1 haplotypes whose frequencies were larger than 0.03, only DQA1*05-DQB1*03 revealed a significant association with a strong effect on the vaccine booster, as presented in **Supplementary Table S8**. Specifically, compared with noncarriers, the estimated OR of being a nonresponder to the first booster for carriers of DQA1*05-DQB1*03 was 0.31 (95% CI = 0.10–0.96, p = 0.042), and the p value of Fisher’s exact test was 0.014 (q = 0.091).

## Discussion

In this case–control study, we enrolled a cohort of healthy, nonsmoking, and non-betel nut-chewing college students who had received a complete cycle of the HBV vaccine during infancy. Approximately 78% had a nonprotective residual anti-HBs titer at their health examination (anti-HBs titer < 10 mIU/mL). A significant proportion (18.7%) of participants who had received the HBVac during infancy did not have protective anti-HBs titers (<10 mIU/mL) after receiving one HBV booster, indicating that they may have lost immunological memory against the HBsAg. A further 2.8% of participants had a nonreactive anti-HBs level after being administered the three-dose HBV booster complete cycle. We observed that DPA1*01 was the significant protective allele, and DPA1*02 was the key risk in the long-term immunological response to the primary neonatal HBVac. In addition, the response to the HBV booster vaccination was significantly influenced by the protective effect of DPB1*03 allele and risk effect of DPB1*05 and DQB1*02 alleles. Participants with DQB1*02 variants were less likely to develop an adequate immunological response after a complete cycle of three HBV-vaccine boosters.

We also observed that DPA1*02-DPB1*05 and DPA1*01-DPB1*03 were associated with a weaker and stronger effect on the response to the vaccine booster, respectively. These observations could be explained by the allelic associations of DPB1*05 and DPB1*03 with response to the vaccine booster. However, we observed that haplotype DQA1*05-DQB1*03 exhibited a significant association with a stronger effect on the vaccine booster, but DQA1*05 and DQB1*03 were not individually associated with vaccine booster response.

HLA class II gene products are expressed in antigen-presenting cells to bind antigenic peptides derived from exogenous proteins and present them to CD4^+^ helper cells. Allelic variations in HLA molecules can affect their ability to alter T-cell recognition and consequently initiate or suppress a cascade of adaptive immune responses to clear HBV. A large-scale genomewide study involving the Taiwan Biobank identified HLA class II variants associated with chronic HBV infection. HLA-DPA1*02:02 and HLA-DPB1*05:01 were discovered to be associated with chronic HBV, and HLA-DRB1*13:02, HLA-DQA1*01:02, and HLA-DQB1*06:09 were negatively associated with HBV chronicity (31). The researchers reported that HLA-DPB1*05:01 exhibited low-affinity binding to peptide epitopes emanated from the HBV nucleocapsid protein and was associated with a low probability of HBV clearance. Hence, they suggested that different HLA variants may induce various nucleocapsid-specific CD4^+^ helper cell responses to HBV (31).

The antigen-binding affinity of HLA class II molecular variations has also been associated with responsiveness to the HBV vaccine and may share a similar mechanism with the HBV infection (22, 24, 31, 34). In the Taiwan Biobank study, HLA-DPA1*02 and HLA-DPB1*05, which have relatively low-affinity binding to peptide epitopes derived from the HBV nucleocapsid protein, were associated with chronic HBV (31), similar to our HBV nonresponse. Conversely, HLA-DQA1*01 and HLA-DQB1*06 were associated with HBV chronicity negatively (31), which is also in congruence with our findings on persistent anti-HBs levels. A systematic meta-analysis reported that DQB1*06 was associated with a significant increase in anti-HBs response to the HBV vaccine, with a pooled OR of 2.34; DQB1*02 (pooled OR = 0.27), however, was correlated with nonresponsiveness to the vaccine (22). DQB1*06 had both a relatively high specificity (75.6%) and low sensitivity (40.2%) in predicting the antibody response to the HBV vaccine. DQB1*02 exhibited low sensitivity (31.2%) and specificity (34%) in the sensitivity and specificity tests of HLA class II variants. In our study, DQB1*06 alleles correlated with the likelihood of sustaining a long-term immunological response in participants who had received primary infantile HBVac, and only five participants remained nonresponders after completing a three-dose cycle of the HBV-vaccine booster. Furthermore, we noted a significant nonresponse association with HBV booster failure for DQB1*02 alleles (p = 0.0087), suggesting that Han people who possess DQB1*02 variants have a strongly increased likelihood of an undetectable postbooster anti-HBs titer, although our sample is small because only 2.8% of vaccinated individuals were categorized as nonresponders.

In 2013, Wu et al. conducted a nested case–control study to evaluate associations of the genetic variants of HLA-DPB1 with single-dose booster response to HBVac. The scholars demonstrated that HLA-DPB1*05 and HLA-DPB1*09 alleles correlated significantly with the likelihood of an undetectable postbooster anti-HBs titer, and HLA-DPB1*02, *03, *04, and *14 alleles significantly reduced this likelihood (34). They further reported that HLA-DPA1*01 correlated with low ORs of an undetectable anti-HBs titer, and HLA-DPA1*02 and HLA-DPB1*05 correlated with high ORs (24). Our study indicated that DPA1*01 and DPA1*02 are protective and risk alleles, respectively, for the long-term immunological response to the primary infantile HBV vaccination; DPB1*03 and DPB1*05 correlated with protective and risk responses to the HBV booster vaccination, respectively. The discrepancy between the research findings of Wu et al. and our results may be attributable the different ethnicity of the populations included in our studies. Although we both administered the same 20 µg/mL/vial Engerix-B recombinant HBV vaccine for our HBV booster vaccinees, our study only comprised people of Han ethnicity. Wu et al. may have included Taiwanese indigenous groups of Malayo-Polynesian ethnicity (34) because their study was conducted in a location with an approximately 28% indigenous population (35). This ethnicity-based variation is supported by the 2007 study of Wang et al., which reported that indigenous people had a significantly increased likelihood of nonresponse to the booster HBV vaccination compared with their Han Chinese counterparts in Taiwan (36). Despite our small sample size, we speculate that DPB1*03 and DPB1*05, and not DPB1*02, *04, and *14, are the strongest influential factors of response to the HBV vaccination in terms of DPB1 because of their significant association with an anti-HBs titer in spite of the limited sample population.

The WHO has stated that no evidence indicates the requirement for an extra dose of HBVac if a person has received a complete series of HBVac in the past (11). However, in particular risk groups such as the HCP (12), HLA-typing analysis and more immunogenic vaccines may be offered to promote a stronger immune response in low- and nonresponders. A Japanese study reported that specific HLA-DRB1-DQB1 haplotypes exhibited different responses to two commonly used HBV vaccines (Heptavax-II and Bimmugen) (37). The US Immunization Action Coalition-recommended Heplisav-B, which contains an adjuvant that activates the toll-like receptor 9 agonist to enhance the immune response, can be considered for use during the revaccination of HCPs with a serum anti-HBs of less than 10 mIU/mL and who received a complete series of HBV vaccines from a different manufacturer in the past (38).

Our study had several limitations. First, the vaccination history of many participants was obtained verbally from their parents without the presentation of a vaccination record. Maternal HBV status was also not obtained in this study. As a result, the actual percentage of hepatitis B immune globulin administered during the neonatal period could not be assessed in this study. However, in 1987, 3 years after HBVac began in Taiwan, the coverage rate of the neonatal HBVac was already as high as 92.4%, with 79.3% of all neonates receiving the full HBV vaccine cycle (39). In addition, students with anti-HBc positivity were excluded from this study to avoid the possibility of occult HBV infection. Hence, the reported receipt of HBV vaccines in our population was likely accurate. Second, to our knowledge, the frequencies of the studied alleles are unknown, and no large-scale studies of HBV reactivation programs among adults have been conducted in the overall Taiwanese population. Hence, different responses would be difficult to speculate on at the population level. Further large-scale studies are required to provide more concrete data. Nevertheless, the results of this study may be incorporated into other research to broaden the understanding of the immune response of the HBV vaccine among young Taiwanese adults (24, 34, 40). Third, as a result of limited funding, we did not test HLA-DR, despite extensive studies both domestically and internationally (20, 23, 26–29) reporting a strong linkage disequilibrium between HLA-DR and -DQ alleles. A weak response was associated with HLA-DRB1*03 and HLA-DRB1*07, and these alleles were in linkage disequilibrium with DQB1*02 in other studies (27, 28). Fourth, extreme obesity is a factor influencing immune response to the HBV vaccine (17). Unfortunately, we did not collect weight and height records for our participants. However, Wang et al. indicated that a high body mass index was nonsignificantly associated with a decreased likelihood of nonresponse to the HBV-vaccine booster among Taiwanese adolescent participants (36). Finally, we did not have data on the initial anti-HBs response to the primary HBV vaccination during our participants’ infancy and cannot therefore report on the kinetic change in their primary vaccination response during adolescence.

## Conclusion

Our study determined that HLA class II DP and DQ alleles were associated with HBV vaccination response in Taiwan. HLA-DPA1*01 exhibited a protective effect on the long-term response to the neonatal HBV vaccination. By contrast, HLA-DPA1*02 posed a risk for long-term response to the primary HBV vaccination during infancy. In addition, HLA-DPB1*05 and HLA-DQB1*02 were associated with nonresponse in the HBV booster effect, but HLA-DPB1*03 had a protective effect on the HBV booster vaccination. A larger-scale study is warranted to corroborate our findings.

## Data Availability Statement

The original contributions presented in the study are included in the article/**Supplementary Material**. Further inquiries can be directed to the corresponding author.

## Ethics Statement

All participants signed an informed consent form, and this study was approved by the Joint Institutional Review Board of Taipei Medical University (N201511029) and the Institutional Review Board of National Cheng Kung University Hospital (B-ER-106-006). The patients/participants provided their written informed consent to participate in this study.

## Author Contributions

Conceptualization, W-CW, Y-SL, C-CY, and F-HS. Funding, F-HS. Designed and performed experiments, Y-SL, Y-FC, C-TS, J-SW, and F-HS. Formal analysis, W-CW, and F-HS. Writing the original draft, W-CW, Y-SL, and F-HS. Review and editing the manuscript, W-CW, Y-SL, Y-FC, C-CY, C-TS, J-SW, and F-HS. All authors contributed to the article and approved the submitted version.

## Funding

This research was supported by grants from the Taiwan Ministry of Science and Technology (MOST 105-2314-B-038-038 and MOST 106-2314-B-038-052-MY3) and Taipei Medical University and Hospital (105TMU-TMUH-19).

## Conflict of Interest

The authors declare that the research was conducted in the absence of any commercial or financial relationships that could be construed as a potential conflict of interest.

## Publisher’s Note

All claims expressed in this article are solely those of the authors and do not necessarily represent those of their affiliated organizations, or those of the publisher, the editors and the reviewers. Any product that may be evaluated in this article, or claim that may be made by its manufacturer, is not guaranteed or endorsed by the publisher.
